# Help4Mood: avatar-based support for treating people with major depression in the community

**Published:** 2012-06-15

**Authors:** Christopher Burton, Maria Klara Wolters, Antoni Serrano Blanco, Aurora Szentagotai, Jenny Ure, Claudia Pagliari, Brian McKinstry

**Affiliations:** University of Edinburgh, UK; University of Edinburgh, UK; Parc Sanitari Sant Joan de Déu, Spain; Babes-Bolyai University, Romania; University of Edinburgh, UK; University of Edinburgh, UK; University of Edinburgh, UK

**Keywords:** depression, avatar-based interaction, user requirements gathering

## Abstract

**Background:**

The Help4Mood consortium, comprising partners from Scotland, Spain, Romania, and Italy, aims to develop a system for supporting the treatment of people with major depressive disorder in the community. The Help4Mood system consists of three parts: (1) A Personal Monitoring System that collects activity and sleep data; (2) a Virtual Agent (avatar) that interacts with the patient in short, structured sessions that involve mood checks, psychomotor tests, and brief therapy-related exercises; (3) a Decision Support System that controls the interaction between user and Virtual Agent, extracts relevant information from the monitoring data, and produces reports for clinicians. In this paper, we report the results of focus groups that were conducted to gather user requirements for Help4Mood. These involved two core stakeholder groups, patients with depression and clinicians.

**Aims and objectives:**

We invited comments on all aspects of system design, focusing on the nature and intensity of monitoring; the interaction between the Virtual Agent and patients; and the support patients and clinicians would wish to receive from Help4Mood.

**Methods:**

Ten focus groups were conducted in Scotland, Spain, and Romania, one each with patients and 2–3 each with psychiatrists, clinical psychologists, and psychiatric nurses. Following a presentation of a sample Help4Mood session, the discussion was structured using a set of prompts. Group sessions were transcribed; data were analysed using framework analysis.

**Results:**

Regarding the overall Help4Mood system, participants raised three main issues, integration with treatment; configurability to support local best practice; and affordability for health services. Monitoring was discussed in terms of complexity, privacy in shared spaces, and crisis. Clinicians proposed physiological, mood, and neuropsychomotor variables that might be monitored, which would yield a complex picture of the patient’s state. Obtrusiveness (of monitors) and intrusiveness (to routines and environment) were raised as important barriers. Patients felt that Help4Mood should provide them with tailored resources in a crisis; clinicians were clear that Help4Mood should not be used to detect acute suicide risks. Three main design characteristics emerged for the Virtual Agent—ease of use, interaction style, and humanoid appearance. The agent should always react appropriately, look and behave like a good therapist, and show positive or neutral emotion. Core functions of the decision support system were characterized by the themes adaptation, informing treatment, and supporting clinician-patient interaction. The decision support system should adapt the patient’s session with the Virtual Agent in accordance with the patient’s mood and stamina. Monitoring data should be presented as a one-page summary highlighting key trends to be discussed with the patient during a consultation.

**Conclusions:**

Consulting with patients and clinicians showed that Help4Mood should focus on tracking recovery in the community in a way that informs and supports ongoing treatment. The design of the Virtual Agent will be crucial for encouraging adherence to the Help4Mood protocol. To ensure uptake of the system, Help4Mood needs to be flexible enough to fit into different service delivery contexts.

